# Involvement of ammonia metabolism in the improvement of endurance performance by tea catechins in mice

**DOI:** 10.1038/s41598-020-63139-9

**Published:** 2020-04-08

**Authors:** Shu Chen, Yoshihiko Minegishi, Takahiro Hasumura, Akira Shimotoyodome, Noriyasu Ota

**Affiliations:** Biological Science Research, Kao Corporation, 2606 Akabane, Ichikai-machi, Haga-gun Tochigi, 321-3497 Japan

**Keywords:** Molecular biology, Nutrition

## Abstract

Blood ammonia increases during exercise, and it has been suggested that this increase is both a central and peripheral fatigue factor. Although green tea catechins (GTCs) are known to improve exercise endurance by enhancing lipid metabolism in skeletal muscle, little is known about the relationship between ammonia metabolism and the endurance-improving effect of GTCs. Here, we examined how ammonia affects endurance capacity and how GTCs affect ammonia metabolism *in vivo* in mice and how GTCs affect mouse skeletal muscle and liver *in vitro*. In mice, blood ammonia concentration was significantly negatively correlated with exercise endurance capacity, and hyperammonaemia was found to decrease whole-body fat expenditure and fatty acid oxidation–related gene expression in skeletal muscle. Repeated ingestion of GTCs combined with regular exercise training improved endurance capacity and the expression of urea cycle–related genes in liver. In C2C12 myotubes, hyperammonaemia suppressed mitochondrial respiration; however, pre-incubation with GTCs rescued this suppression. Together, our results demonstrate that hyperammonaemia decreases both mitochondrial respiration in myotubes and whole-body aerobic metabolism. Thus, GTC-mediated increases in ammonia metabolism in liver and resistance to ammonia-induced suppression of mitochondrial respiration in skeletal muscle may underlie the endurance-improving effect of GTCs.

## Introduction

Exercise-induced fatigue is an important concern in sports, exercise, and rehabilitation because it is unavoidable and its early onset can hinder people from accomplishing their goals. Exercise-induced fatigue is commonly assessed as endurance capacity, based on the assumption that time to exhaustion correlates with the force generating capacity of muscle, and it is attributed to multiple factors, including the accumulation of fatigue metabolites, depletion of muscle glycogen, decrease of muscle pH, increase of muscle temperature, and production of inflammatory cytokines^1,2^. The complexity of the mechanisms underlying the development of exercise-induced fatigue makes it difficult to find approaches to mitigate its effects.

Ammonia is a ubiquitous waste product of the metabolism of nitrogenous compounds, including amino acids and proteins, and has long been considered both a central and peripheral factor in the onset of exercise-induced fatigue^3^. During exercise, ammonia is produced via deamination of adenosine monophosphate^4,5^ and the breakdown of branched-chain amino acids in skeletal muscle^6,7^. The production of ammonia increases with increasing intensity and duration of exercise^8–10^. Although the ammonia fatigue theory is not new, there remains a lack of conclusive evidence proving the roles of ammonia in the onset and development of exercise-induced fatigue.

Recent evidence from exercise and disease studies has provided new insights into the role of ammonia during exercise. For example, it has been reported that ammonia activates phosphofructokinase and inhibits the oxidation of pyruvate to acetyl coenzyme A, which hinders the supply of adenosine triphosphate to skeletal muscle^5^. In addition, studies in cirrhosis patients have revealed that hyperammonaemia impairs protein synthesis and increases autophagy^11,12^, induces mitochondrial dysfunction due to cataplerosis of α-ketoglutarate, and lowers adenosine triphosphate content in muscle cells, which may contribute to skeletal muscle fatigue^12,13^. Since plasma concentrations of ammonia during exercise often reach or exceed those found in liver disease patients^14,15^, these toxic effects may also be transiently induced during exercise, resulting in muscle fatigue. However, it remains unknown whether these observed localised effects of hyperammonaemia disrupt energy metabolism throughout the whole body.

In mammals, ammonia is detoxified by being metabolised to urea via the urea cycle in the liver. In mice, supplementation with amino acids that are components of the urea cycle, such as citrulline, arginine, and ornithine, has been reported to improve endurance by decreasing blood ammonia concentration during exercise^16,17^. Similarly, in humans, supplementation with citrulline has been reported to increase cycling time-trial performance^18^. Thus, activation of the urea cycle may be one way of reducing fatigue and improving exercise endurance capacity.

Flavonoids such as nobiletin^19^, naringin^20^, chrysin^21^, and fisetin^22,23^ have been shown to improve ammonia resistance and up-regulate urea cycle enzymes^19,20,22^; however, whether this up-regulation is effective for lowering exercise-induced hyperammonaemia remains unknown. One group of flavonoids, the green tea catechins (GTCs), have been shown to have several health-promoting impacts, including anti-cancer effects, hepatoprotective effects, anti-diabetic effects, and anti-obesity effects^24^; however, the impact of GTCs on ammonia metabolism remains to be examined.

Murase *et al*. previously reported that GTCs improve exercise endurance in mice^25,26^ and humans^27^. Murase *et al*. also reported that GTCs increase fatty acid translocase/CD36 mRNA expression and fatty acid β-oxidation in skeletal muscles, resulting in stimulation of lipid utilisation during exercise^25,26^, which is considered a likely fundamental mechanism of the endurance-improving effect of GTCs. These findings prompted us to investigate whether dietary GTCs improve ammonia-resistance, since ammonia attenuates mitochondrial function in muscle^13^. That is, we hypothesised that GTCs improve exercise endurance by increasing ammonia resistance in muscle cells. Thus, in the present study we examined the effect of hyperammonaemia on whole-body energy metabolism, and the relationship between ammonia metabolism and the improvement of exercise endurance by GTCs.

## Results

### Changes in blood ammonia concentration during and after exercise, and the relationship between blood ammonia concentration and total running time

**Figure 1:**
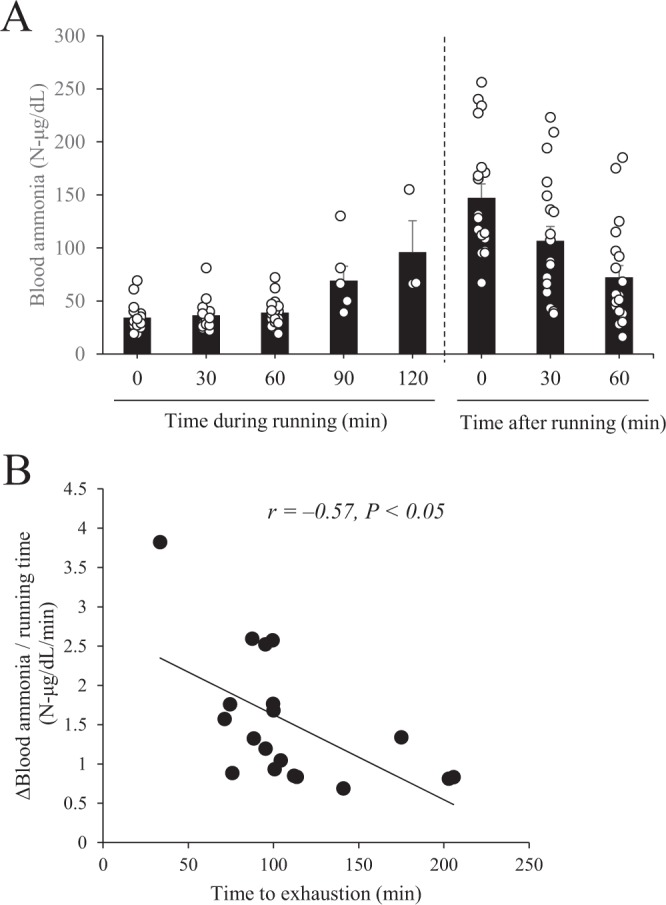
Relationship between the rate of increase of blood ammonia concentration and the time during and after running in BALB/c mice. (**A**) Blood ammonia concentration was measured during and after running at the indicated time points. (**B**) Pearson’s correlation coefficient (*r*) was obtained to estimate the linear correlation between the rate of increase of blood ammonia concentration and time during and after running (95% CI: −0.814 to −0.158). Data are presented as mean ± s.e.m.; *n* = 19.

Blood ammonia concentration gradually increased over time as running continued, and then gradually decreased after the end of running (Fig. 1A). The rate of increase of blood ammonia concentration was significantly negatively correlated with total running time, which was measured as an index of exercise endurance capacity (Fig. 1B; *r* = −0.57, *P* < 0.05).

### Effect of hyperammonaemia on energy metabolism in mice

**Figure 2:**
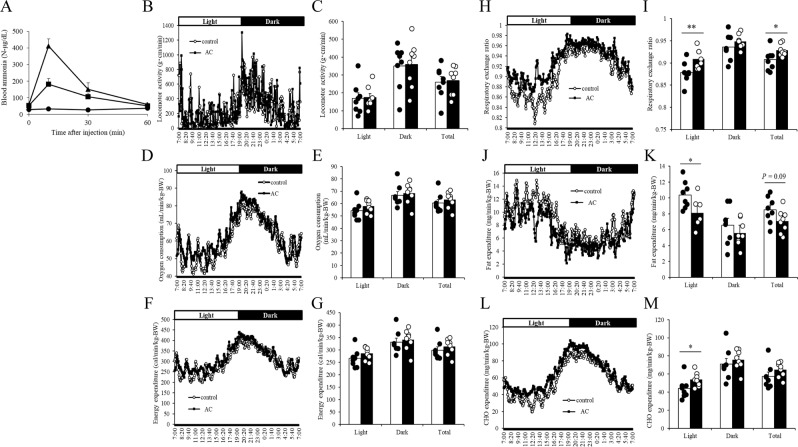
Effect of hyperammonaemia on energy metabolism in mice. (**A**) Mice were intraperitoneally administered 0 (circles), 100 (squares), or 200 mg/kg-BW (triangles) ammonium chloride, and blood ammonia was measured at 0, 10, 30, and 60 min after administration. (B–M) Ammonium chloride solution (AC, 100 mg/kg-BW) was administered intraperitoneally to BALB/c mice 3 times a week for 4 weeks. Energy expenditure in terms of locomotor activity (**B,C**), oxygen consumption (**D,E**), and respiratory exchange ratio (**F,G**) were measured by indirect calorimetry. Fat expenditure (**H,I**), carbohydrate (CHO) expenditure (**J,K**), and total energy expenditure (**L,M**) were calculated by using equations described previously^38^. Mean values for each time point are shown. Data are presented as mean ± s.e.m.; *n* = 3 mice per group (A), *n* = 8 mice per group (B–M). *P < 0.05, **P < 0.01 (Mann–Whitney U test).

Next, we examined the changes in energy metabolism induced by hyperammonaemia by intraperitoneally injecting mice with 0, 100, or 200 mg/kg body weight (BW) of ammonium chloride (Fig. 2A). Ammonium chloride dose-dependently increased blood ammonia concentration, and the maximum blood ammonia concentration in the mice administered 100 mg/kg-BW ammonium chloride, which was reached at 10 min after administration, was comparable with that induced by exercise (Fig. 1A). Next, we administered mice 100 mg/kg-BW of ammonium chloride 3 times a week for 4 weeks to induce chronic hyperammonaemia and then measured energy metabolism during the day and night by indirect calorimetry. In the hyperammonaemic mice, we observed no significant differences in locomotor activity (Fig. 2B,C), oxygen consumption (Fig. 2D,E), or energy expenditure (Fig. 2F,G) during the day or night compared with those in non-treated control mice; however, we did observe significantly increased respiratory exchange ratio (Fig. 2H,I), significantly decreased fat expenditure (Fig. 2J,K), and significantly increased carbohydrate expenditure (Fig. 2L,M) during the day.

### Effect of hyperammonaemia on the expression of lipid metabolism–related genes in mouse skeletal muscle

**Figure 3:**
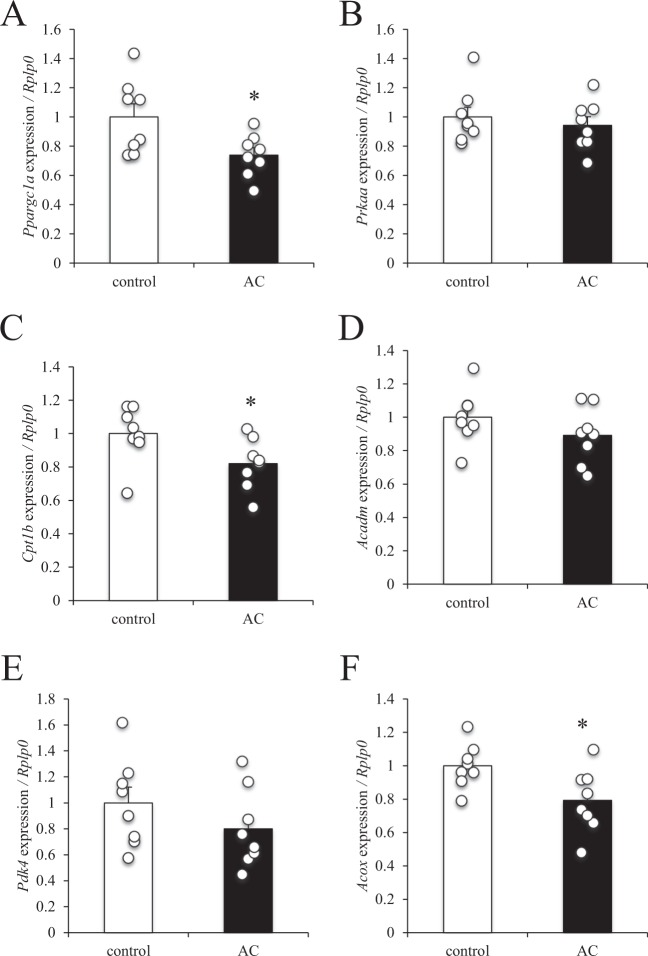
Effect of hyperammonaemia on lipid metabolism–related gene expression in mouse skeletal muscle. Ammonium chloride solution (AC, 100 mg/kg-BW) was administered intraperitoneally to BALB/c mice 3 times a week for 4 weeks, after which the gastrocnemius muscle was resected. mRNA expression levels of (**A**) *Ppargc1a*, (**B**) *Prkaa*, (**C**) *Cpt1b*, (**D**) *Acadm*, (**E**) *Pdk4*, and (**F**) *Acox* were examined using quantitative RT-PCR and normalised to the expression of *Rplp0*. Data are presented as mean ± s.e.m.; *n* = 8. *P < 0.05 (Mann–Whitney U test).

To evaluate the effect of chronic hyperammonaemia on lipid metabolism in skeletal muscle, gene expression in the mouse gastrocnemius muscle was examined. The expression levels of peroxisome proliferator-activated receptor gamma coactivator 1-α (*Ppargc1a*), carnitine palmitoyltransferase 1b (*Cpt1b*), and acyl-CoA oxidase (*Acox*) were significantly decreased in the hyperammonaemic mice compared with control mice (Fig. 3A,C,F), whereas the expression of adenosine monophosphate-activated protein kinase (*Prkaa*), medium-chain acyl-CoA dehydrogenase (*Acadm*), and pyruvate dehydrogenase kinase 4 (*Pdk4*) remained unchanged (Fig. 3B,D,E).

### Effect of hyperammonaemia on mitochondrial respiration in muscle cells and skeletal muscle tissue

**Figure 4:**
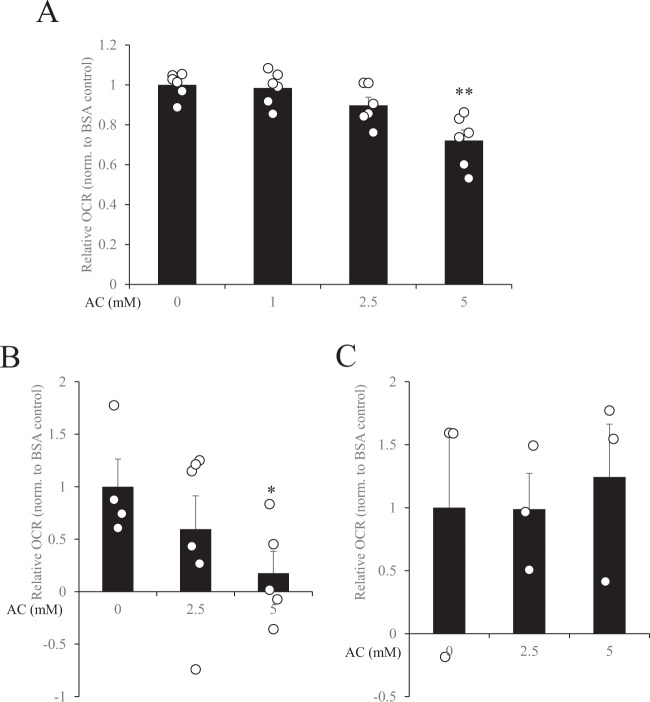
Effect of hyperammonaemia on mitochondrial respiration in C2C12 myotubes and skeletal muscle tissues. (**A**) C2C12 myoblasts were differentiated into myotubes for 4 days in DMEM supplemented with 2% horse serum, and oxygen consumption rate (OCR) in response to 100 µM palmitate stimulation was measured in the presence or absence of ammonium chloride (AC; 0, 1, 2.5, or 5 mM). (**B**) Soleus and (**C**) extensor digitorum longus were resected, and OCR in response to 100 µM palmitate stimulation was measured in the presence or absence of AC (0, 2.5, or 5 mM). Data were normalized (norm.) to the BSA control (AC 0 mM). Data are presented as mean ± s.e.m.; *n* = 6 (**A**); n = 3–5 (**B,C**). *P < 0.05, **P < 0.01 vs. AC 0 mM (ANOVA with Dunnett’s multiple comparison test).

To evaluate the effect of hyperammonaemia on fatty acid oxidation in muscle cells and skeletal muscle tissues, we evaluated mitochondrial respiration by using an extracellular flux analyser to measure oxygen consumption rate (OCR) in response to palmitate stimulation. Administration of 5 mM ammonium chloride significantly suppressed OCR compared with control in C2C12 myotubes (Fig. 4A) and soleus (Fig. 4B), but not in extensor digitorum longus (Fig. 4C).

### GTCs enhanced endurance capacity and decreased liver ammonia in mice

**Figure 5:**
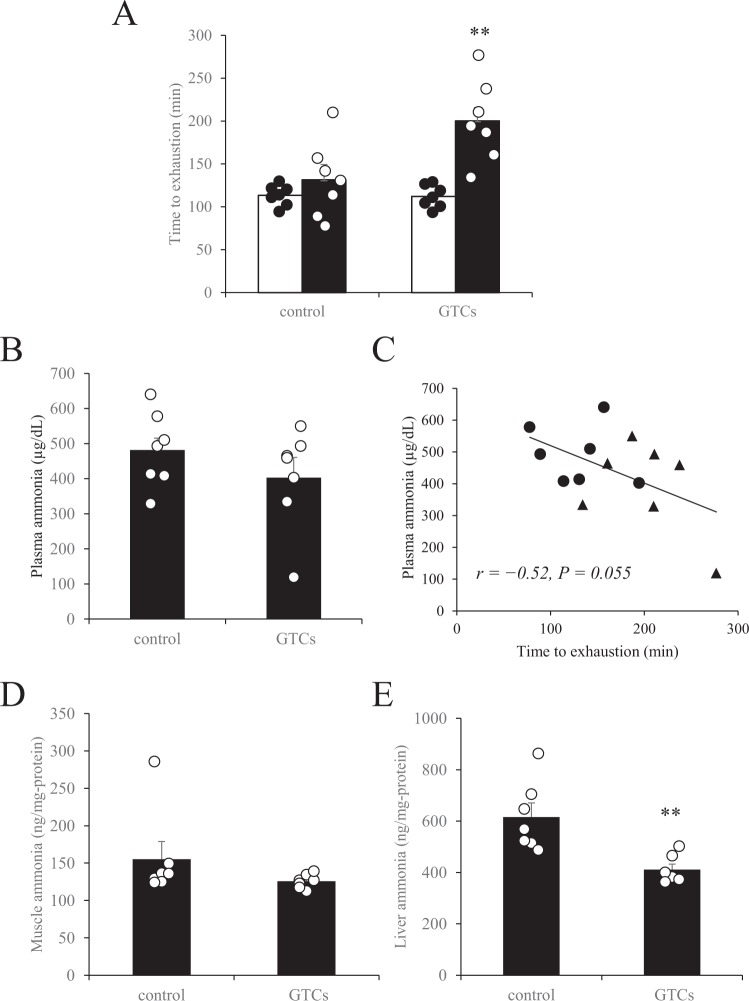
Effect of the intake of green tea catechins (GTC) on exercise endurance performance and plasma, skeletal muscle, and liver ammonia concentrations in mice. (**A**) After 4 weeks of exercise training, running time to exhaustion was measured to evaluate endurance performance. Open bars, pre-exercise training; closed bars, post-exercise training. Two days after the endurance evaluation, mice were subjected to a 60-min treadmill running test, immediately after which serum (**B**), muscle (**D**), and liver ammonia concentrations (**E**) were determined. (**C**) Pearson’s correlation coefficient (*r*) was obtained to estimate the linear correlation between exercise endurance capacity (95% CI: −0.825 to 0.009) (A) and plasma ammonia concentration determined after 60 min of running (**B**). Circles, control mice; triangles, mice administered GTCs. Data are presented as mean ± s.e.m.; *n* = 7. **P < 0.01 (Wilcoxon signed-rank test (**A**), Mann–Whitney U test (**B,D,E**)).

Four weeks of continuous oral administration of GTCs and training intervention significantly improved exercise endurance in mice, whereas no improvement was observed in the mice subjected to training alone (control group) (Fig. 5A). After 60 min of running, though plasma ammonia remained unchanged between GTCs and control group (Fig. 5B), plasma ammonia concentration was tended to negatively correlated with maximum running time (Fig. 5C, *r* = −0.52, *P* = 0.055). Administration of GTCs did not change the amount of ammonia in the gastrocnemius muscle (Fig. 5D), but it did significantly decrease the amount of ammonia in the liver (Fig. 5E).

### Effect of GTCs on the expression of genes encoding urea cycle enzymes in mouse liver

**Figure 6:**
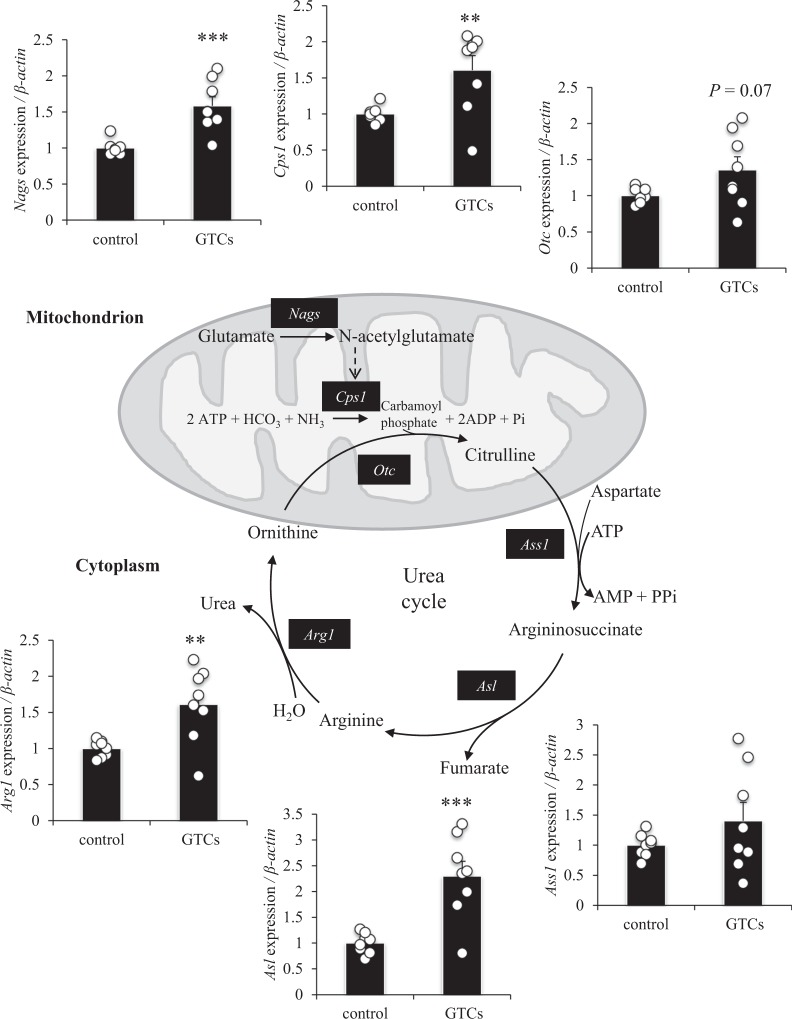
Effect of the intake of green tea catechins (GTCs) on genes encoding urea cycle enzymes in mouse liver. After 8 weeks of exercise training, the liver was dissected, and mRNA expression levels of *Nags*, *Cps1*, *Otc*, *Ass1*, *Asl*, and *Arg1* were examined by qRT-PCR and normalised to the expression of β-actin. Data are presented as mean ± s.e.m.; *n* = 8. **P < 0.01, ***P < 0.001 (Mann–Whitney U test).

Eight weeks of continuous ingestion of GTCs and exercise training significantly improved endurance running time in mice, whereas training alone had no significant effect (Supplementary Fig. S1 online). In addition, ingestion of GTCs significantly increased the expression of *Nags*, *Cps1*, *Asl*, and *Arg1*, compared with control; the expression of *Otc* tended to increase (*P* = 0.07) and the expression of *Ass1* remained unchanged (Fig. 6).

### Effect of GTCs on gene expression of urea cycle enzymes in mouse primary liver cells

**Figure 7:**
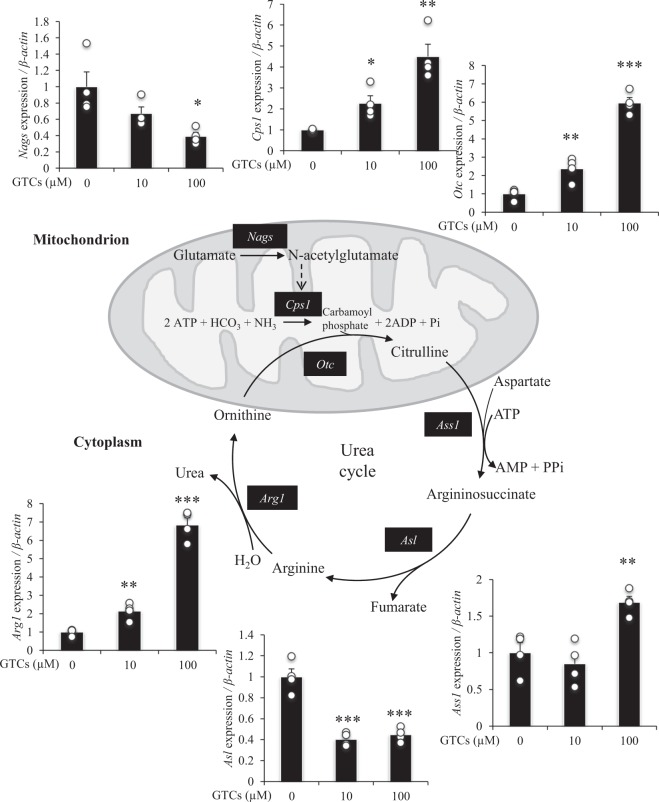
Effect of green tea catechins (GTCs) on the expression of urea cycle genes in mouse liver cells. Primary liver cells were cultured in DMEM (foetal bovine serum-, arginine-, and leucine-free) for 24 h in the presence or absence of GTCs (0, 10, 100 µM). Total RNA was extracted, and the mRNA expression of *Nags*, *Cps1*, *Otc*, *Ass1*, *Asl*, and *Arg1* were examined by qRT-PCR and normalised to the expression of β-actin. Data are presented as mean ± s.e.m.; *n* = 4. *P < 0.05, **P < 0.01, ***P < 0.001 (ANOVA with Dunnett’s multiple comparison test).

In primary mouse liver cells, GTC treatment significantly increased the expression of *Cps1*, *Otc*, *Ass1*, and *Arg1*, but significantly decreased the expression of *Nags* and *Asl*, compared with control (Fig. 7).

### Effect of pre-incubation with GTCs on mitochondria respiration in muscle cells

**Figure 8:**
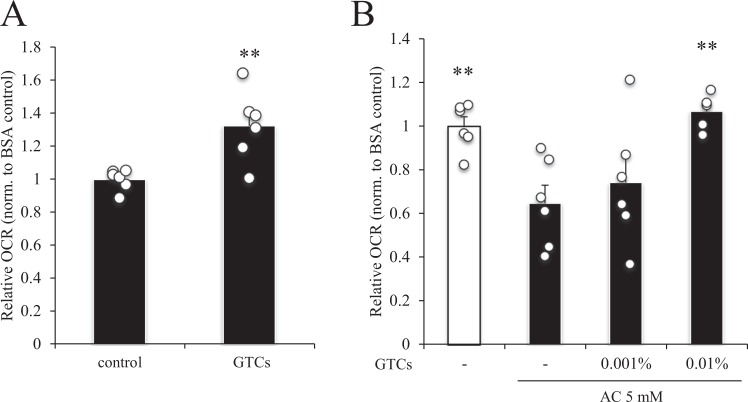
Effect of green tea catechins (GTCs) on mitochondrial respiration in C2C12 myotubes. C2C12 myoblasts were differentiated into myotubes for 4 days in DMEM supplemented with 2% horse serum. Differentiated myotubes were treated with 0.01% GTCs (**A**) or 0.001 or 0.01% GTCs (**B**) for 24 h in DMEM supplemented with 2% horse serum, and oxygen consumption rate (OCR) was measured in the absence (A) or presence (**B**) of 5 mM ammonium chloride (AC). Relative OCR values were normalized (norm.) to BSA control. Data are presented as mean ± s.e.m.; *n* = 6. (**A**) **P < 0.01 (Mann–Whitney U test). (**B**) **P < 0.01 vs. GTCs (−)/5 mM AC (ANOVA with Dunnett’s multiple comparison test).

Finally, we evaluated the effect of GTCs on fatty acid oxidation in muscle cells by measuring OCR. Pre-incubation of the muscle cells with 0.01% GTCs significantly increased OCR in C2C12 myotubes compared with control (Fig. 8A). Exposure to 5 mM ammonium chloride significantly decreased OCR in the C2C12 myotubes, and exposure to 0.01%, but not 0.001%, GTCs rescued the ammonium chloride-induced reduction in OCR (Fig. 8B).

## Discussion

This study had three major findings. First, hyperammonaemia decreased whole-body aerobic metabolism and reduced fat expenditure and lipid metabolism–related gene expression in skeletal muscle in mice. Second, continuous ingestion of GTCs combined with exercise training increased endurance capacity and the expression of urea cycle–related genes in mice. Finally, hyperammonaemia suppressed mitochondrial respiration, but this suppression was rescued by pre-incubation with 0.01% GTCs in C2C12 myotubes.

For almost a century, ammonia production during exercise has been suggested to lead to both peripheral and central fatigue^3^. However, the ammonia fatigue theory is often not considered because of the lack of strong empirical evidence. To clarify the relationship between ammonia production and exercise performance, we subjected mice to an endurance performance test, and during and after the test we monitored blood ammonia concentration. We found that blood ammonia concentration gradually increased with increasing running time, which is consistent with the results of Banister *et al.*^8^ who showed that blood ammonia production increases in response to prolonged exercise in humans. We also found a significant negative relationship between blood ammonia concentration and exercise endurance capacity, which is consistent with a previous report^28^. In the present study, we did not find a correlation between blood ammonia concentration at the exhaustive point and exercise endurance capacity (data not shown). Thus, we speculate that a strong determinant of exercise endurance capacity is the rate of increase of blood ammonia concentration during exercise, rather than the maximum concentration achieved. In other words, the balance between the production and elimination of ammonia from the body is a crucial factor for ensuring endurance performance.

The effects and underlying mechanism of ammonia-induced fatigue remain controversial, especially with regard to peripheral effects. Recent studies in cirrhosis patients have revealed that hyperammonaemia impairs protein synthesis and induces mitochondrial dysfunction in skeletal muscle^11–13^. However, whether hyperammonaemia disturbs whole-body energy metabolism has not been examined. To evaluate the effect of hyperammonaemia on whole-body energy metabolism, we injected mice with ammonium chloride solution 3 times a week for 4 weeks. After each injection, blood ammonia concentration was raised to the same level induced by endurance exercise. After 4 weeks, respiratory exchange ratio and carbohydrate expenditure were significantly increased, fat expenditure was significantly decreased, and oxygen consumption and energy expenditure were the same in the ammonium chloride-administered mice as in the control mice. These results indicate that hyperammonaemia may induce a switch in the mode of energy utilization from fatty substrates to glucose substrates. Analysis of skeletal muscle from the mice administered ammonium chloride revealed that the gene expression levels of *Ppargc1a*, *Cpt1b*, and *Acox* were significantly decreased. Since these genes are related to mitochondrial and peroxisomal respiration in skeletal muscle, fatty acid–induced aerobic respiration might be impaired in hyperammonaemic mice, which would contribute to a whole-body decrease of fat utilisation. Davuluri *et al*. have reported that hyperammonaemia results in dysfunction of skeletal muscle mitochondria due to cataplerosis of α-ketoglutarate and lower adenosine triphosphate content^13^. Our *in vitro* study also showed that ammonium chloride dose-dependently decreased palmitate-induced OCR, which indicates that there was a decline of fatty acid–induced mitochondrial respiration. Taken together, these results suggest that hyperammonaemia causes acute suppression of mitochondrial respiration, and that chronic hyperammonaemia can supress the gene expression of lipid metabolism–related enzymes and thus reduce fat utilisation in skeletal muscle. Hyperammonaemia induced by ammonium chloride injection has been reported to induce substantial sensations of fatigue in humans^29^; however, whether ammonium chloride injection affects human metabolism or endurance capacity needs further investigation.

Our *ex vivo* study showed that exogenous ammonia has different effects on different types of muscle fibre type. Exposure to 5 mM ammonium chloride decreased the OCR in soleus but had no effect in the extensor digitorum longus, indicating that the soleus, which mainly contains slow-twitch muscle fibres, is more sensitive to ammonium chloride stimulation than the extensor digitorum longus, which mainly contains fast-twitch muscle fibres. Takeda *et al*. have reported that ammonia transporters are more abundant in slow-twitch muscle fibres^28^. Thus, more exogenous ammonia may be incorporated into the muscle fibres in soleus compared with in extensor digitorum longus. However, muscle itself generates ammonia during exercise, and adenosine monophosphate deaminase, which produces ammonia, is reported to be more abundant in fast-twitch muscle fibres than in slow-twitch muscle fibres. Therefore, although fast-twitch muscle fibres have less ammonia transporters compared with slow-twitch muscle fibres, fast-twitch muscle fibres may still be exposed to more ammonia compared with slow-twitch muscle fibres. Considering that we added ammonia to the outside of the muscle fibres, our results are limited to the *ex vivo* context and so may not reflect the *in vivo* environment of muscle fibres. An *in vivo* model is needed to elucidate the precise effects of ammonia on the different types of muscle fibres.

Two flavonoids have been reported to decrease blood ammonia by increasing the expression of urea cycle enzymes^19–23^. However, whether this increased expression improves exercise endurance remains unknown. GTCs have been shown to improve exercise endurance by enhancing lipid metabolism in skeletal muscle^25,26^, although the involvement of ammonia in this context is yet to be examined. Because ammonia attenuates mitochondrial respiration in muscle, we examined the potential of GTCs to increase ammonia resistance. We found that 4 weeks of continuous oral administration of GTCs and exercise training significantly improved maximum endurance running time in mice. In addition, blood ammonia concentration after 60 min of running was negatively correlated with running time and plasma ammonia tended to decrease in GTC mice. Furthermore, although GTC intake significantly decreased liver ammonia after 60 min of running, no change in muscle ammonia was observed. These results indicate that continuous GTC intake may be beneficial for increasing exercise endurance capacity and ammonia metabolism during exercise. However, as we only examined plasma, muscle, and liver ammonia at one time-point during running, a multipoint examination and determination of the total ammonia reduction over the whole exercise period are needed. At least our gene expression analysis revealed that the expressions of *Nags*, *Cps1*, *Asl*, and *Arg1*, which are key enzymes in the urea cycle, were increased in mice administered GTCs, which goes some way to explain the effect GTCs had on lowering blood ammonia concentration during exercise.

To evaluate whether the GTCs had a direct effect on urea cycle genes, we directly stimulated primary mice liver cells with GTCs. In a preliminary experiment, treatment with 100 µM GTCs for 24 h did not cause cytotoxicity in primary mice liver cells (data not shown). We found that the expression levels of *Cps1*, *Otc*, *Ass1*, and *Arg1* were increased by exposure to GTCs, whereas those of *Nags* and *Asl* were decreased. Recent studies have reported that urea cycle genes are transcriptionally regulated by clock-controlled genes, such as Klf15 and C/EBPβ, and thus their expression oscillates with the circadian rhythm^19,30,31^. Also, the ureagenic capacity of the urea cycle has been shown to be up-regulated in response to metabolic challenge^32^. The fact that the *in vitro* condition lacks the metabolic regulations found *in vivo* likely explains why our *in vitro* and *in vivo* results differ. In addition, our *in vivo* studies were combined with exercise, which induces dynamic changes in metabolite availability that cannot be accurately reflected in *in vitro* studies. Since the intermediates of the urea cycle are in part cross-linked with those of the tricarboxylic acid cycle^33,34^, the reason that the expression levels of *Nags* and *Asl* were decreased *in vitro* needs further investigation with a focus on the metabolites of the urea and tricarboxylic acid cycles. At least the expression of *Cps1*, the rate-limiting enzyme of the urea cycle and the amount of blood ammonia, was up-regulated dose-dependently by GTCs, which might indicate a direct positive effect of GTCs on ammonia metabolism.

Finally, we examined the direct effect of GTCs on fatty acid oxidation in muscle cells by measuring OCR. We found that exposure of muscle cells to GTCs dose-dependently improved their OCR and rescued the suppression of OCR induced by hyperammonaemia. Since ammonia attenuates mitochondrial respiration in muscle^13^, continuous intake of GTCs may improve ammonia resistance in muscle cells. However, whether intake of GTCs can improve ammonia-induced decrease of whole-body fat expenditure *in vivo* needs to be analysed in a future study.

Together, our data suggests that GTCs increase ammonia metabolism in liver and increase resistance to ammonia-induced suppression of mitochondrial respiration in skeletal muscle, and that these two effects contribute to the endurance-enhancing effect of GTCs. However, the present study has some limitations. First, the relationship between ammonia and other peripheral muscle fatigue factors is not clear. We found that muscle glycogen tended to increase compared with control mice after 60 min of running in mice administered GTCs, but this finding was not significant (data not shown) and muscle lactate was found to remain the same as control mice in the present study. Since these factors are known to contribute to endurance capacity, time-course analysis of muscle glycogen, lactate, ammonia and other metabolites such as H^+^ or inflammatory cytokines will be needed. Second, antioxidants, including GTCs are known to increase endurance capacity and energy expenditure through their antioxidant effects and stimulation of lipid utilization during exercise^25,35,36^. Our finding that GTCs rescued the suppression of OCR induced by hyperammonaemia may be explained by the antioxidant effects of GTCs, since ammonium chloride induces oxidative stress and mitochondrial dysfunction in muscle cells^13^. However, the effect of GTCs on liver ammonia metabolism is unknown, and this is the first time a study has shown that GTCs-mediated increases in the expression levels of urea cycle genes in the liver. Since endurance exercise is known to induce oxidative stress in the liver^37^, the antioxidant effect of GTCs might protect the liver from oxidative stress; however, the relationship between ammonia metabolism and antioxidant effect of GTCs remains to be elucidated. Third, central fatigue was not examined in the present study. Since exercise-induced fatigue includes both peripheral and central aspects, and ammonia is considered a central fatigue factor^10^, the role of GTCs on central fatigue warrants further investigation.

In conclusion, the present results indicate that enhanced ammonia metabolism in liver, and enhanced resistance to ammonia-induced attenuation of mitochondrial respiration in skeletal muscle, may contribute to the exercise endurance-enhancing effect of GTCs. Our study demonstrates a novel physiological function and mechanism of GTCs with relation to ammonia metabolism, and our findings will be useful for the development of improved treatments for fatigue and hyperammonaemia-related disorders.

## Materials and Methods

### GTCs

For the *in vivo* studies we used polyphenon 70 S (Mitsui Norin Co. Ltd., Shizuoka, Japan), which is a commercially available GTC mixture purified from 100% tea leaves. The polyphenon 70 S we used contained 80.9% catechins as follows: epigallocatechin gallate (32.3%), epigallocatechin (20.0%), epicatechin gallate (8.8%), epicatechin (7.9%), gallocatechin (6.1%), catechin (1.8%), gallocatechin gallate (3.2%), and catechin gallate (0.8%). The remaining 19.1% was water (<8%), carbohydrates (<10%), proteins (<1%), lipids (<1%), and ash (<1%).

For the *in vitro* studies, a mixture of GTCs was prepared and its composition was analysed as described previously^25^. The catechins in this mixture were epigallocatechin gallate (34.84%), epigallocatechin (34.43%), epicatechin gallate (9.75%), epicatechin (8.54%), gallocatechin (6.72%), catechin (2.93%), gallocatechin gallate (1.69%), and catechin gallate (1.1%).

GTC concentrations were calculated using the average catechin molecular weight (374.8) and specific gravity (1.05 g/mL).

### Animals and diets

All animal experiments were conducted at the Experimental Animal Facility of Kao Tochigi Institute (Tochigi, Japan) and were approved by the Animal Care Committee of Kao Corporation (Tokyo, Japan). All experiments were performed in accordance with relevant guidelines and regulations.

### Experiment 1

Male BALB/c mice (12-weeks old; Charles River Laboratories Japan, Kanagawa, Japan) were maintained in a room with controlled temperature and relative humidity (23 ± 2 °C, 55 ± 10%, respectively) and a 12-h light–dark cycle. Mice were subjected to treadmill running, during which running endurance capacity was measured (see *Evaluation of endurance performance*). Blood ammonia was quantified with a portable handheld blood ammonia meter (PocketChem BA PA-4140; Arkray, Inc., Kyoto, Japan) at 0, 30, 60, 90, and 120 min after the start of running, and at 0, 30, 60 min after the end of running. During the experimental period, mice were fed CE-2 (CLEA Japan, Tokyo, Japan) and water was provided *ad libitum*.

### Experiment 2

Male BALB/c mice (30-weeks old; Charles River Laboratories) were maintained as described in Experiment 1. Mice were intraperitoneally administered ammonium chloride (Wako Pure Chemical Industries, Ltd., Osaka, Japan) solution (100 mg/kg-BW) 3 times a week for 4 weeks^23^. The same amount of saline was administered to negative control animals. During the experimental period, mice were fed CE-2 (CLEA Japan) and water was provided *ad libitum*. After 4 weeks, energy metabolism was analysed by indirect calorimetry (see *Indirect calorimetry analysis*); then the mice were anesthetised with isoflurane (Abbott Japan, Tokyo, Japan) and the gastrocnemius muscle was resected and stored at −80 °C.

### Experiment 3

Male BALB/c mice (6-weeks old; Charles River Laboratories) were maintained as described in Experiment 1. At 8 weeks, initial running endurance capacity was measured by allowing the mice to run and then recording time to exhaustion; then the mice were divided into two groups (*n* = 7) based on average running time with a body weight equivalent. Mice were fed the control diet (Supplementary Table S1 online) and water was provided *ad libitum*. During the experimental period, mice were subjected to a 30-min treadmill running exercise (20 m/min) 5 days a week for 4 weeks. Before exercise, mice were orally administered 0.2 g/kg-BW GTCs or distilled water as negative control. At the end of the 4 weeks, endurance capacity was again evaluated. Two days after the endurance evaluation, the mice were subjected to 60-min treadmill running at 25 m/min on an 8° incline and then anesthetised with isoflurane immediately after running. Blood samples were collected from the abdominal vein into capillary blood collection tubes (CAPIJECT with EDTA-2Na; Terumo Medical Co., Tokyo, Japan) and maintained on ice until plasma preparation. After centrifugation at 3500 × *g* for 15 min at 4 °C, plasma samples were stored at −80 °C. The gastrocnemius muscle and liver were also resected and stored at −80 °C.

### Experiment 4

Male BALB/c mice (6-weeks old; Charles River Laboratories Japan) were maintained as described in Experiment 1. At 8 weeks, initial running endurance capacity was measured by allowing the mice to run and then recording time to exhaustion; then the mice were divided into two groups (*n* = 8) based on average running time with a body weight equivalent. Mice were fed either the control or 0.5% GTC diet (Supplementary Table S1 online) and water was provided *ad libitum*. During the experimental period, mice were subjected to a 30-min treadmill running exercise (20 m/min on an 8° incline) 4 days a week for 8 weeks and endurance capacity was again evaluated. Two days after endurance measurement, mice were anesthetised with isoflurane and the liver was resected and stored at −80 °C.

### Evaluation of endurance performance

A 10-lane motorized rodent treadmill (MK-680; Muromachi Kikai, Tokyo, Japan) with an 8° incline was used to evaluate endurance capacity with the following program: 10 m/min (6 min) → 12 m/min (2 min) → 14 m/min (2 min) → 16 m/min (2 min) → 18 m/min (2 min) → 20 m/min (2 min) → 22 m/min (2 min) → 24 m/min (2 min) → 26 m/min (2 min) → 28 m/min until exhaustion

### Indirect calorimetry analysis

In Experiment 2, energy metabolism was analysed by using an ARCO-2000 magnetic-type mass spectrometric calorimeter (ARCO System, Chiba, Japan). During the measurement, mice were allowed *ad libitum* access to water and feed (CE-2). Locomotor activity, oxygen consumption (VO_2_), and carbon dioxide exhalation (VCO_2_) were measured, and respiratory exchange ratio, fat expenditure, carbohydrate expenditure, and total energy expenditure were calculated by using equations described previously^38^. The measurement of energy metabolism was carried out for 72 h and the data from the middle 24-h period were used to avoid using the variable data at the beginning and end of the measurement period.

### Acquisition and culture of primary liver cells

Primary liver cells were acquired as previously reported^39^. Briefly, male BALB/c mice (13–18-weeks old; Charles River Laboratories) were anesthetised with isoflurane, and Liver Perfusion Medium (GIBCO, Rockville, MD) followed by Liver Digest Medium (GIBCO) were perfused through the portal vein. Then, the liver was resected and cells were filtrated and washed with DMEM (Sigma-Aldrich Co., LCC., Tokyo, Japan) three times and seeded in a 24-well plate at 10^5^ cells/well in DMEM. After 24 h, the cells were treated with 10–100 µM GTCs in DMEM (foetal bovine serum-, arginine-, and leucine-free) for another 24 h and then collected for gene expression analysis.

### Quantitative RT-PCR

Total RNA was extracted by using an RNeasy Mini Kit (QIAGEN K.K., Tokyo, Japan). Reverse transcription was performed by using a High-Capacity cDNA Kit with random primers and a Veriti 96-Well Thermal Cycler (Life Technologies Japan, Ltd., Tokyo, Japan). qRT-PCR was performed by using a TaqMan probe (Life Technologies) on an ABI ViiA 7 Real-Time PCR System (Life Technologies). Data were normalised to acidic ribosomal protein P0 (*Rplp0*) or β-actin mRNA content.

### Measurement of ammonia

Blood ammonia was quantified with a portable handheld blood ammonia meter (PocketChem BA PA-4140). Plasma, muscle, and liver ammonia were quantified with an Ammonia/Ammonium Assay Kit (Abcam, Cambridge, MA). Muscle and liver ammonia were normalised to total protein measured with a BCA Protein Assay Kit (Thermo Scientific, Rockford, IL).

### Analysis of fatty acid oxidation in myotubes and intact skeletal muscle tissue

#### Cell culture and treatments

C2C12 myoblasts (European Collection of Authenticated Cell Cultures, Salisbury, UK) were seeded at 1000 cells/well in XF96 cell-culture microplates and maintained in DMEM containing 10% foetal bovine serum and penicillin–streptomycin (Life Technologies Japan Ltd., Tokyo, Japan) at 37 °C in 5% CO_2_ in air. Once the cells reached confluence they were differentiated into myotubes for 4 days in DMEM supplemented with 2% horse serum (Life Technologies) and penicillin–streptomycin at 37 °C in 5% CO_2_ in air. Differentiated myotubes were treated with 0.001 or 0.01% GTCs for 24 h in DMEM supplemented with 2% horse serum, and OCR was measured as described below.

#### Fatty acid oxidation analysis in differentiated myotubes

To examine fatty acid oxidation in differentiated myotubes, a XF96 Flux Analyzer (Agilent Technologies Japan, Ltd., Tokyo, Japan) was used and the analysis was conducted in accordance with the manufacturer’s protocol for the extracellular flux assay. Basal respiration of differentiated myotubes was monitored, and then 0.1 mM palmitate–bovine serum albumin (BSA) (Sigma-Aldrich) or fatty acid–free BSA was injected onto the cells automatically from the injection port. At the same time, ammonium chloride (1, 2.5, 5 mM) or Seahorse XF DMEM medium (Agilent Technologies) as the negative control was injected to evaluate the effect of hyperammonaemia on myotube mitochondrial respiration. Relative OCR was calculated by subtracting the OCR value of the cells exposed to fatty acid–free BSA (control) from that of the cells exposed to palmitate–BSA.

#### Fatty acid oxidation analysis in intact skeletal muscle

Analysis of fatty acid oxidation in intact skeletal muscle was performed as previously reported^40^. Briefly, male BALB/c mice (12–16 weeks; Charles River Laboratories) were anesthetised with isoflurane, and intact skeletal muscles (soleus and extensor digitorum longus) were resected and cut into 2 × 2 × 1 mm sections. The muscle pieces were then put into a XF^e^24 Islet Capture Microplate (Agilent Technologies) and covered with capture screens. After 1-h pre-incubation in Seahorse XF DMEM medium, basal respiration of the skeletal muscles was determined, and then 0.1 mM palmitate–BSA or fatty acid–free BSA was added, as described above. At the same time, ammonium chloride (2.5 or 5 mM) or negative control (Seahorse XF DMEM medium) was added to evaluate the effect of hyperammonaemia on mitochondrial respiration of the skeletal muscles. Relative OCR was calculated as described above.

### Statistics

Data are presented as mean ± s.e.m. The Mann–Whitney U test for independent samples and the Wilcoxon signed rank test for dependent samples were used for single comparisons. One-way ANOVA followed by Dunnett’s post-hoc test was used for multiple comparisons. Associations between two parameters were analysed using Pearson’s correlation coefficient. The threshold for significance was *P* < 0.05. Data were analyzed using Microsoft Excel 2010 (Microsoft Corp., Redmond, WA) and GraphPad Prism6 (GraphPad software Inc., La Jolla, CA).

## Supplementary information

Supplementary information.
