# Supplementary material for: Involvement of ammonia metabolism in the improvement of endurance performance by tea catechins in mice
Source: Sci Rep. 2020 Apr 8;10:6065. doi: 10.1038/s41598-020-63139-9 (PMC7142105; doi:10.1038/s41598-020-63139-9)
Supplement: Supplementary file 1 — Supplementary information. [file 41598_2020_63139_MOESM1_ESM.pdf]

TITLE PAGE

Involvement of ammonia metabolism in the improvement of endurance performance by tea catechins in mice

Shu Chen, Yoshihiko Minegishi, Takahiro Hasumura, Akira Shimotoyodome and Noriyasu Ota

Ingredient	Control (%)	0.5% GTCs (%)
Potato starch	55.5	55.0
Corn oil	10.0	10.0
Casein	20.0	20.0
Cellulose	8.1	8.1
Mineral mixture	4.0	4.0
Vitamin mixture	2.2	2.2
Methionine	0.2	0.2
GTCs	0.0	0.5

Supplementary Table S1. Compositions of the experimental diets.
GTCs, green tea catechins.

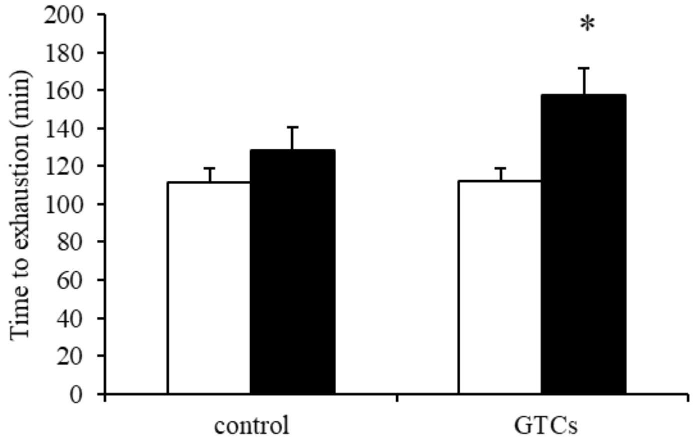

Supplementary Figure S1. Effect of the intake of green tea catechins (GTC) on exercise endurance performance in mice. After 8 weeks of exercise training, running time to exhaustion was measured to evaluate endurance performance. Open bars = pre-exercise training; closed bars = post-exercise training. Data are presented as mean ± s.e.m.; $n = 8$. * $P < 0.05$ (Mann–Whitney U test).
